# Cumulated time to chart closure: a novel electronic health record-derived metric associated with clinician burnout

**DOI:** 10.1093/jamiaopen/ooae009

**Published:** 2024-02-08

**Authors:** Madhura Shah, Sofia De Arrigunaga, Leah S Forman, Matthew West, Susannah G Rowe, Rebecca G Mishuris

**Affiliations:** Boston University Aram V. Chobanian & Edward Avedisian School of Medicine, Boston, MA 02118, United States; Department of Ophthalmology, Bascom Palmer Eye Institute, University of Miami Medical School, Miami, FL 33136, United States; Biostatistics and Epidemiology Data Analytics Center, Boston University School of Public Health, Boston, MA 02118, United States; Department of Biostatistics, Harvard T.H. Chan School of Public Health, Boston, MA 02115, United States; Office of Equity, Vitality and Inclusion, Boston University Medical Group, Boston, MA 02118, United States; Wellness and Professional Vitality, Boston Medical Center, Boston, MA 02118, United States; Department of Ophthalmology, Boston University Aram V. Chobanian & Edward Avedisian School of Medicine, Boston, MA 02118, United States; Digital, Mass General Brigham, Somerville, MA 02145, United States; Department of General Internal Medicine, Brigham and Women’s Hospital, Boston, MA 02115, United States

**Keywords:** burnout, electronic health records, documentation burden, physician wellbeing

## Abstract

**Objective:**

We sought to determine whether average cumulated time to chart closure (CTCC), a novel construct to measure clinician workload burden, and electronic health record (EHR) measures were associated with a validated measure of burnout.

**Materials and methods:**

Physicians at a large academic institution participated in a well-being survey that was linked to their EHR use data. CTCC was defined as the average time from the start of patient encounters to chart closure over a set of encounters. Established EHR use measures including daily total time in the EHR (EHR-Time8), time in the EHR outside scheduled hours, work outside of work (WOW_8_), and time spent on inbox (IB-Time8) were calculated. We examined the relationship between CTCC, EHR use metrics, and burnout using descriptive statistics and adjusted logistic regression models.

**Results:**

We included data from 305 attendings, encompassing 242 432 ambulatory encounters (2021). Among them, 42% (128 physicians) experienced burnout. The median CTCC for all clinicians was 32.5 h. Unadjusted analyses revealed significant associations between CTCC, WOW_8_, IB-Time8, and burnout. In a final adjusted model, only CTCC remained statistically significant with an odds ratio estimate of 1.42 (95% CI, 1.00-2.01).

**Discussion:**

These results suggest that CTCC is predictive of burnout and that purely measuring duration of interaction with the EHR itself is not sufficient to capture burnout.

**Conclusion:**

Workload burden as manifested by average CTCC has the potential to be a practical, quantifiable measure that will allow for identification of clinicians at risk of burnout and to assess the success of interventions designed to address burnout.

## Background and significance

Electronic health records (EHRs) have been adopted widely with the aim of improving health care efficiency, patient safety, and quality of care. However, EHR use has also demonstrably contributed to clinician stress and burnout—which in turn may jeopardize patient safety and other outcomes the EHR proposes to improve.^1^^,^^2^ Studies have shown that for every 8 h of scheduled patient time, ambulatory physicians spend >5 h on the EHR, much of that performing tasks that are perceived as a clerical burden that interferes with time spent with patients.^2–4^ This time burden faced by clinicians has been implicated in rising clinician occupational distress and burnout.^5^

To address these issues, in 2016 the 21st Century Cures Act 2016 began requiring that EHR vendors monitor and report on the usability of their products to maintain their certification.^6^ The development of standard and objective EHR use measures has been proposed as an essential component to this program.^7^ In this regard, the American Medical Association (AMA) “Joy in Medicine (TM) System Recognition Program” provides a roadmap for health systems to assess and intervene on drivers of burnout, including EHR use. One of the six pillars of assessment, “Efficiency of Practice Environment,” encompasses measuring time spent on the EHR and implementing interventions based on this audit.^8^

A 2023 scoping review of vendor- and investigator-derived measures of EHR use identified nine observational studies examining the association between EHR use and burnout, turnover, or stress.^9^ However, the exact mechanism of interaction between the individual and the EHR that leads to stress remains uncharacterized. Understanding this association is critical in proposing interventions that will reduce burnout. This lack of causal understanding is due, in part, to variations in the definition of active EHR use and burnout itself.

A recent study exploring the relationship between vendor-derived EHR use metrics and ambulatory care physician turnover provided additional insight into the limitations of existing metrics.^10^ Some EHR use metrics (total EHR time, work outside of scheduled clinical hours, encounter note documentation time, active time on inbox, and teamwork for orders) were directionally associated with likelihood of departure. Paradoxically, less time spent on the EHR was associated with greater likelihood of departure. This counterintuitive association of total time spent on the EHR and departure suggests there are other factors at play not captured in the existing EHR use metrics. Aligned with these findings, a recent paper highlighted that metrics that seek to characterize the interaction between physicians and the EHR largely focus on active EHR time and concluded that documentation burden remains poorly defined.^10^

Recognizing both the value and the limits of previously described EHR use metrics to predict clinician well-being, our research team attributes these limitations in part to the fact that existing measures may not fully reflect the totality of workload burden in the context of clinician preferences and life circumstances. Additionally, assessing clinician well-being using validated measures of burnout is a time-intensive effort for both health systems and clinicians.

These gaps emphasize the need for a real-time metric to assess work burden which would be: (1) directly correlated with clinician occupational well-being and thus serve as a proxy for burnout, (2) resistant to bias due to FTE (full time equivalence), (3) considerate of individual work practice preferences, (4) accessible and effective in early identification of clinicians at risk of burnout, and (5) difficult to artifactually skew or “game” without resulting in actual improvement in burnout.

## Objective

**Figure 1.**
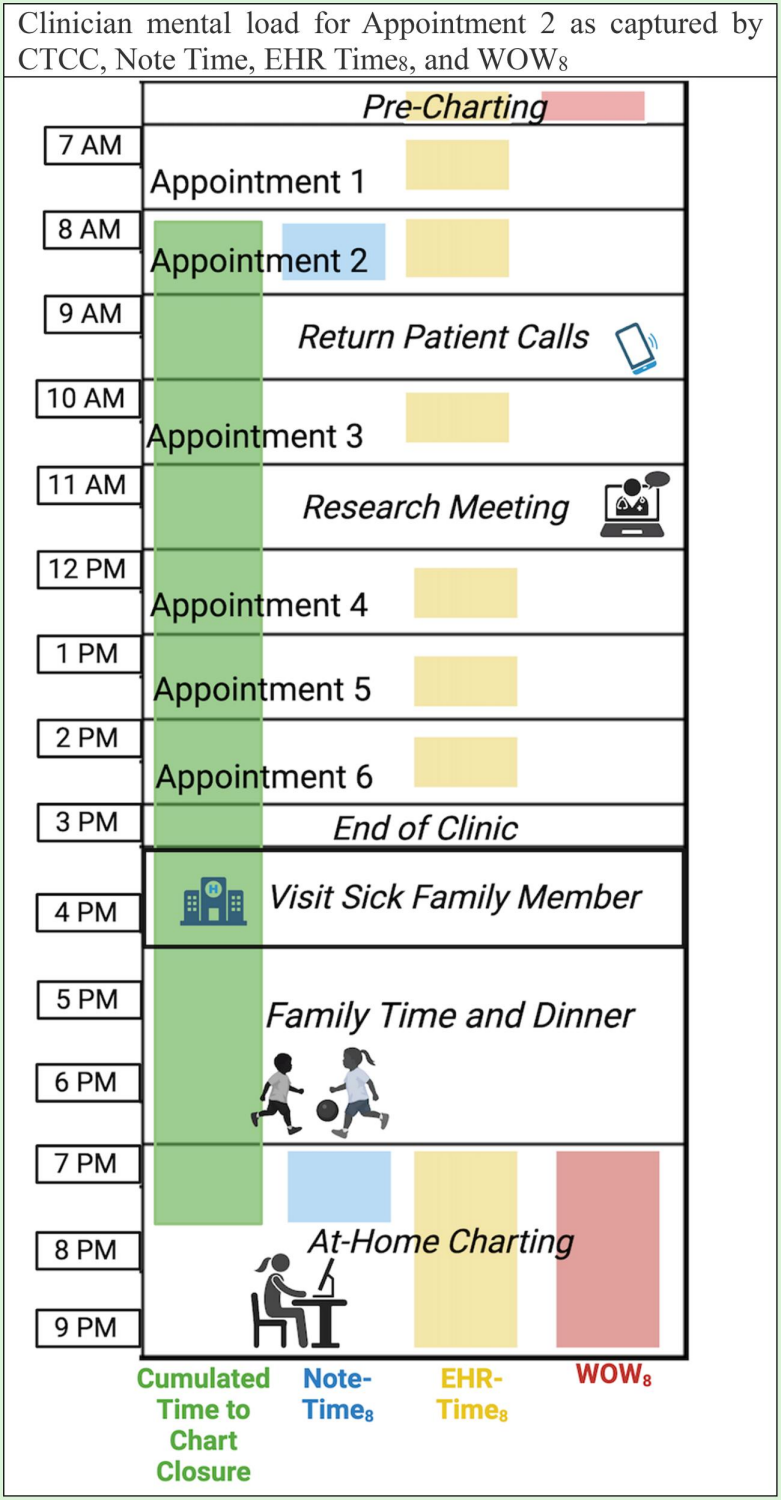
Comparison of a clinician’s clinical mental load in one day as captured by Chart Closure Time, Note Time, EHR-Time_8_, and WOW_8_. Chart Closure Time is longer than the Note-Time_8_ for the note associated with “Appointment 2” as it considers the delay between the end of the appointment and when the encounter is closed, including outside of clinic obligations such as caregiving, research, and family activities during which the mental load of the clinical encounter and remaining clinical documentation work is still carried. Neither EHR-Time_8_ nor WOW_8_ account for this additional dimension of a clinician’s professional and personal life and its interaction with their clinical responsibilities.

We propose a new metric, cumulated time to chart closure (CTCC), to further characterize the balance of clinician job demands and resources, which materializes in the way the EHR is used. In this construct, workload burden is manifested in the mean CTCC as defined as the mean amount of time elapsed between the start of a clinical encounter and the completion of all required documentation for that visit, for a set of encounters over a period of time. CTCC, therefore, not only accounts for the clinician’s EHR-based work by including time spent using the EHR, but also considers their routines, and work and non-work responsibilities outside of their interaction with the EHR by including time elapsed between the EHR interactions required to complete any given clinical encounter (Figure 1). We posit that the totality of these demands on the clinician’s time will contribute to time elapsed from the start of the encounter to the completion of required documentation. We further hypothesize that burnout further contributes to the mental load of these demands and may result in longer CTCC, as well as a long CTCC may be an indicator of burnout due to the mismatch between resources and demands, and mental load that it represents.

We define CTCC (per encounter) as the clock time elapsed between when a physician begins a patient encounter and when the encounter is closed (with all required documentation complete) in the EHR. This is not limited to time spent in the EHR, and simply describes the difference in timestamps between encounter start and documentation completion, thus accounting for other demands on the clinician’s time and mental capacity (Figure 1). Workload burden is therefore defined as the mean of CTCC across a set of encounters.

Considering the multifaceted and non-linear impact of EHR on care delivery—from documentation to safety—this interplay between clinical work, non-clinical work, personal life, and EHR use should be considered in investigating burnout. For example, when a clinician undergoes a personal challenge, perhaps to care for an ailing family member after a day of clinical responsibilities, additional time may lapse between the initiation and completion of a note. Workload burden, the average CTCC for their clinical encounters, would increase while other EHR use metrics would not be affected. CTCC is also sensitive to long periods of inactivity in the EHR, such as when notes are left incomplete over multiple days. Workload burden and CTCC would be increased in the case of an understaffed clinic, where the clinician is burdened by administrative tasks throughout the clinic day. He or she may be left with more outstanding notes and less time to complete them at the end of the day, increasing CTCC but not other metrics such as overall time spent on the EHR or in specific tasks such as note writing.

Here, we describe the relationship between CTCC and a validated measure of clinician burnout.^1^ We also describe the relationships between previously described EHR-use metrics and burnout.

## Methods

This retrospective cross-sectional study was approved by the Institutional Review Board at Boston Medical Center (BMC).

Between April and June of 2021, the Boston University Medical Group (BUMG) of Boston Medical Center, Boston, Massachusetts, conducted an administrative survey through an independent third-party survey administrator to inform organizational efforts to improve professional fulfillment and well-being among clinical faculty. All physician and advanced practice professional (physician assistants and nurse practitioners) members of the clinically active medical staff with an on-campus appointment were invited to participate via email (*n* = 1287), and 930 completed the survey. Up to nine reminders were sent to non-respondents. Participation was voluntary. Responses from partially completed surveys were included if they contained at least 75% of completed items for each measure. Survey response rate was 72%.

The Professional Fulfillment Index (PFI) well-being survey is a standardized, validated tool for holistic assessment of burnout and professional fulfillment with a supplemental questionnaire for intent to leave current institution.^1^ Clinicians were determined to be burned out or not according to a score based on responses such as work exhaustion and interpersonal disengagement, on a Likert scale from 0 to 4. A mean of 1.33 or higher on an individual’s response to burnout questions was used as the burnout cutoff based on the PFI’s validation study.^1^

As mentioned above, we define CTCC (per encounter) as the clock time elapsed between when a physician begins a patient encounter and when the encounter is closed (with all required documentation complete) in the EHR—this includes time spent interacting with the EHR itself and elapsed time between these required interactions to account for other demands on the clinician’s time and mental capacity. Workload burden is then expressed as the mean of CTCC across a set of encounters.

Initiation of the patient encounter is marked by the EHR timestamp from Epic’s Clarity database, “visit start.” This time point is triggered in Epic when the encounter is opened in the exam room, vitals are taken, medications or chief complaints are documented, a progress note is started, or a problem list is marked as reviewed, whichever happens first. Encounter closure time is marked in Clarity as well; an encounter can only be closed once all documentation and billing activities are complete. Sensitivity analyses using other potential start timestamps (such as time of appointment and time of physician entry into patient room) showed that differences in the start timestamp used to calculate time to chart closure did not significantly impact the calculation of CTCC nor its association with burnout.

Given the heterogeneity of practice patterns in ambulatory clinics, this pragmatic measure accounts for variation in workflow and work preferences by individual and by specialty. Time to chart closure is aggregated at the clinician level to calculate CTCC, which is defined as the mean time to chart closure over all ambulatory encounters during a specified time window. For example, if a physician has three encounters in a day, with the chart being closed 5 h after start in encounter 1, 3 h after start in encounter 2, and 1 h after start for encounter 3, then their CTCC over this period would be (5 + 3 + 1)/3 = 3 h.

The survey and subsequent EHR use data linkage were conducted after review and approval by the Boston University School of Medicine institutional review board. EHR use data and audit time logs of attending physicians for all of 2021 were extracted from Signal (Epic Systems) and Clarity (Epic Systems), respectively. We included physicians that had worked clinically for at least 30 h in one or more months during that period (*n* = 305).

Previously described EHR use measures^11^ (which only account for time spent directly interacting with the EHR) were retrieved for the cohort from Signal. These include: total time on EHR during and outside of clinic sessions per 8 h of patient scheduled time (EHR-Time_8_), work outside of work which includes time on EHR outside of scheduled patient hours per 8 h of patient scheduled time (WOW_8_), hours on documentation (note writing) per 8 h of scheduled patient time (Note-Time_8_), total time on inbox per 8 h of patient scheduled time (IB-Time_8_), and the percentage of orders with team contribution (TW_ORD_).^3^^,^^4^ We also included a metric which tracks the percent of notes closed on the same day as the encounter (%EncClosed).

Wilcoxon rank sum tests performed with SAS 9.4 software were used to compare unadjusted differences in Workload Burden and each EHR measure between burned out and not burned out physicians. Significance was assessed at α=.05. To evaluate the association between EHR metrics and burnout, we ran logistic regression models with logged versions of primary predictors, adjusting for age, ethnicity, gender, academic rank, and specialty. EHR metrics were log-transformed due to observed non-linearities in their relationship with burnout, as well as being strictly positive. Models were explored in a univariable setting before combining significant predictors into a final model, in each case adjusting for demographics and professional factors. Additional models were fit to explore interaction effects between significant covariates.

## Results

Complete data (both PFI survey data and EHR use data) was available for 305 attending physicians across 242 432 ambulatory encounters between January 1 and October 30, 2021. Most physicians performed their own documentation, with less than one-third using speech to text software and only a handful using in-person scribes.

Of the 305 physicians included, 56% of physicians were female, 69% were under 50 years, and 42% were burned out (Table 1). More than half of the sample (214 physicians, 70%) were medical specialists including primary care physicians; 83 physicians (27%) were surgical specialists, and 8 physicians (3%) were in other procedural specialties.

**Table 1.** Distribution of respondent characteristics among physicians with and without burnout.

	Physicians, *N* (%)			
Variable	Overall (*n* = 305)	No burnout (*n* = 177)	Burnout (*n* = 128)	*P*-value
Age				
Under 39	106 (34.8%)	62 (35.0%)	44 (34.4%)	.2558^a^
Between 40 and 49	104 (34.1%)	55 (31.1%)	49 (38.3%)	
Between 50 and 59	55 (18.0%)	38 (21.5%)	17 (13.3%)	
60 years or more	40 (13.1%)	22 (12.4%)	18 (14.1%)	
Self-identified gender				.7998^a^
Female	170 (55.7%)	96 (54.2%)	74 (57.8%)	
Male	104 (34.1%)	63 (35.6%)	41 (32.0%)	
Other or missing value	31 (10.2%)	18 (10.2%)	13 (10.2%)	
Race/ethnicity		5 (15%)	5 (15%)	
White	181 (59.3%)	110 (62.1%)	71 (55.5%)	.5453^a^
Asian	56 (18.4%)	32 (18.1%)	24 (18.8%)	
African American/Black	13 (4.3%)	8 (4.5%)	5 (3.9%)	
Hispanic/Latino	16 (5.2%)	7 (4.0%)	9 (7.0%)	
American Indian/Alaskan Native	1 (0.3%)	0 (0.0%)	1 (0.8%)	
Native Hawaiian/Pacific Islander	1 (0.3%)	0 (0.0%)	1 (0.8%)	
Unknown/Missing	37 (12.1%)	20 (11.3%)	17 (13.3%)	
Specialty category*				
Medical	214 (70.2%)	119 (67.2%)	95 (74.2%)	.3017^b^
Procedural	8 (2.6%)	4 (2.3%)	4 (3.1%)	
Surgical	83 (27.2%)	54 (30.5%)	29 (22.7%)	
Number of encounters per physician in 2021, median (IQR)	675 (426, 1038)	643 (420, 1139)	724 (448, 996)	.9669^c^

Categorical data are presented as *n* (%) and continuous data are presented as mean (standard deviation).

a Chi-square.

b Fisher’s exact.

c Wilcoxon rank sum.

* Medical: Addiction Medicine, Adolescent Medicine, Allergy, Alternative Medicine, Bariatrics, Cardiac Rehabilitation, Cardiology, Endocrinology, Family Medicine, Gastroenterology, General Internal Medicine, General Practice, Genetics, Geriatric Medicine, Gerontology, Hematology, Oncology, Hospice and Palliative Medicine, Infectious Diseases, Internal Medicine, Neonatology, Nephrology, Neurology, Nutrition, Oncology, Pediatric: Allergy, Cardiology, Endocrinology, Hematology, Infectious Disease, Neurology, Pulmonology, Rheumatology; Preventative Medicine, Primary Care, Psychiatry, Psychology, Pulmonary, Rehabilitation, Rheumatology, and Sports Medicine.

Surgical: Anesthesiology, Breast Surgery, Cardiothoracic Surgery, Colon and Rectal Surgery, Dentistry & Oral/Max Surgery, General Surgery, Neurosurgery, Obstetrics and Gynecology, Ophthalmology, Oral and Maxillofacial Surgery, Orthopedic Surgery, Otolaryngology, Pediatric Surgery, Plastic Surgery, General Surgery, Surgical Oncology, Transplant, Trauma Surgery, Urology, and Vascular Surgery.

Procedural: Audiology, Dermatology, Emergency Medicine, Interventional Radiology, Occupational Therapy, Pediatric: Dermatology, Emergency Medicine, Gastroenterology. Physical Therapy, Podiatry, Radiation Medicine, Radiation Oncology, and Wound Care.

Of the 305 physicians included in the analysis, 177 (58%) were not burned out and 128 (42%) were burned out. The median CTCC for all clinicians was 32.5 h. Median EHR-Time_8_ was 5.2 h, and median WOW_8_ was 1.1 h. In both adjusted and unadjusted analyses, CTCC, WOW_8_, and IB-Time_8_ were significantly associated with burnout. EHR-Time_8_ and Note-Time_8_ were not significantly associated with being burned out in either adjusted or unadjusted analyses (Table 2).

**Table 2.** Association between electronic health record (EHR)-derived metrics and burnout in physicians.

	Median (25th percentile, 75th percentile)			
Variable	Overall (*n* = 305)	No burnout (*n* = 177)	Burnout (*n* = 128)	*P*-value
Cumulated time to chart closure (h)	32.5 (8.43, 96.17)	23.44 (5.76, 85.24)	47.09 (11.2, 111.47)	.0219*
EHR-Time_8_ (h)	5.24 (3.80, 7.32)	4.94 (3.58, 7.06)	5.48 (4.22, 7.54)	.0589
WOW_8_ (h)	1.12 (0.62, 1.92)	0.98 (0.52, 1.76)	1.28 (0.73, 2.08)	.0152*
Note-Time_8_ (h)	2.0 (1.3, 2.8)	1.8 (1.3, 2.6)	2.0 (1.3, 2.9)	.239
IB-Time_8_ (h)	0.62 (0.38, 1.04)	0.56 (0.32, 1.00)	0.71 (0.46, 1.06)	.0129*
TW_ORD_ (%)	10.0 (2.0, 22.0)	11.0 (2.0, 2.5)	9.0 (3.0, 2.0)	.3603
%EncClosed (%)	80.0 (30.0, 100.0)	80.0 (50.0, 100.0)	70.0 (30.0, 90.0)	.1230

* Statistically significant.

To explore interactions between CTCC and other EHR measures in predicting burnout, a final adjusted model was constructed using CTCC, and the two other metrics found to be significant in unadjusted analyses: IB-Time_8_ and WOW_8_. Parameter estimates from this model are shown in Table 3.

**Table 3.** Parameter estimates from final logistic regression model predicting burnout as outcome.

Independent variable	Level^a^	Reference^a^	Odds ratio (95% CI)	*P*-value
CTCC			1.42 (1.00-2.01)	.0473*
WOW_8_			1.04 (0.35-3.14)	.9389
IB-Time_8_			2.02 (0.63-6.47)	.2342
Age			1.02 (0.77-1.34)	.9152
Race/ethnicity	Asian	White	1.33 (0.70-2.55)	.3878
	Under-Represented Minorities		1.50 (0.68-3.32)	.3204
	Unknown/Missing		1.79 (0.60-5.35)	.2967
Gender	Male	Female	1.15 (0.67-1.99)	.6099
	Self-defined or missing		0.73 (0.22-2.39)	.5968
Academic Rank	Associate Professor	Professor	0.57 (0.19-1.77)	.3346
	Assistant Professor		1.05 (0.35-3.13)	.9361
	Instructor		1.06 (0.27-4.12)	.9325
	Missing		0.28 (0.06-1.23)	.0912
Specialty	Medical	Surgical	1.32 (0.74-2.37)	.3470
	Procedural		1.90 (0.38-9.38)	.4315

a For categorial variables.

* Statistically significant.

In this model, CTCC was the only variable significantly associated with an increased risk of burnout, with an odds ratio of 1.42 (95% CI, 1.00-2.01). As EHR predictors were logged prior to model fitting, this can be interpreted as a 10-fold increase in CTCC being associated with an approximate 1.42-fold (42%) increase in the odds of being burned out. Alternatively, a doubling in CTCC is associated with an approximate 1.11-fold (11%) increase in the odds of being burned out.

Due to observed co-linearity between WOW_8_ and IB-Time_8_, additional models were fit to explore the predictive effect of CTCC on burnout in the presence of just one of those measures, adjusted for demographic and professional covariates. In the model with CTCC and WOW_8_ (see Table S1), CTCC had an odds ratio estimate of 1.38 (95% CI, 0.98-1.94) though neither EHR measure met the threshold for statistical significance. In the model with CTCC and IB-Time_8_ (see Table S2), CTCC had an odds ratio estimate of 1.43 (95% CI, 1.02-1.99), and was the only covariate that met the threshold for statistical significance. These results suggest that CTCC is predictive of burnout, with a robust estimate of effect size that is consistently measurable even when adjusting for other EHR measures associated with burnout.

## Discussion

In this retrospective cross-sectional study, we have defined a novel measure of workload burden derived from EHR timestamps, but inclusive of demands on the clinician not directly related to EHR use, and have demonstrated that it is associated with burnout even when adjusting for previously described EHR use metrics. While more work is needed to investigate any causal relationships that may underlie these associations, it is reasonable to suggest that workload burden, as manifest by CTCC, is both a predictor and consequence of burnout. Demands on clinician’s time and mental space, whether job or personal related, for which they do not have the resources—team support, time, mental capacity—to adequately address (ie, workload burden) contribute to burnout. CTCC captures this mismatch of demands and resources, and therefore burnout, in how much time elapses from a clinical visit to completion of the required documentation; the mismatch of holistic demands and resources results in prolonged elapsed time to address documentation requirements. CTCC itself also contributes to demand and mental load—the longer the clinician holds on to the visit in their mind prior to completing documentation, the more it will draw on their mental load to both hold that information as well as recall it to complete documentation. In these ways, CTCC is both a consequence of burnout and contributor to burnout. Having a predictive and easily derivable measure of burnout is critical to identifying opportunities for burnout prevention and the impact of interventions designed to reduce burnout.

Understanding the contributors to burnout is critical to designing and assessing impactful interventions. We believe an imbalance of demands and resources leads to burnout; fluctuating personal and professional factors determine a physician’s ability to carry out their job successfully without creating an imbalance that sacrifices wellness. We posit that this balance of job demands and resources is observable through the length of time over which a physician takes to complete EHR-related work, quantified by CTCC. CTCC reflects the individual’s degree of burnout that results from this imbalance of resources and demands.

The additional dimension of burden of work encompassed by this measure in its assessment of cumulated time of an open note, but not exclusively time spent interacting with the EHR, can further characterize burnout—which results from the lack of capacity of the clinician to complete their clinical work, and the mental load of that incomplete work. CTCC as a measure of workload burden may serve as both a bellwether and proxy for burnout.

The significant association between CTCC, an EHR-derived measure that also accounts for non-EHR-related demands, and burnout suggests its potential as an accessible measure of clinician burden. For instance, a clinician who spends 2 h the evening after a clinic finishing notes will have the same “work outside of work” time as a clinician who spends 2 h finishing notes a week later, but the latter will have a higher workload burden and associated burnout risk. CTCC is a more nuanced measure of the clinician’s workload than just EHR time alone.

Surveys present one way to assess clinician occupational well-being (Maslach, Trockel),^4^ however they can be both expensive and time-consuming (for both the clinician to complete and the organization to administer). Measures derived from EHR log data that are associated with these gold standard burnout assessments may provide a more practical and efficient pathway to understand job sustainability related to the balance between job demands and resources. CTCC is substantially easier to assess than traditional well-being surveys.

Existing EHR use measures offer useful information regarding how clinicians interact with the EHR, yet the totality of a clinician’s experience with the EHR extends beyond these defined variables. For example, a recent study found that physicians with longer notes spend 39% more time in the EHR.^12^ These EHR use metrics do not account for the influence of the clinician’s life circumstances outside of clinical work, nor their preferences for how or when they perform EHR-related work. For example, a clinician may have a high “work outside of work” metric but may prefer charting outside of the hours of 7 am and 7 pm. In this case, the clinician would carry a lower documentation burden while having a high “work outside of work” metric. Sinsky acknowledges these limitations of EHR use measures: “Some vendors and researchers have used clock times, such as 7 pm to 7 am, to establish the window for work outside of work, but this does not account for variability in physician schedules. A physician who utilizes administration days or personal days off to complete documentation and inbox work would appear to have little WOW in this data model.”^13^

The burden embodied by one’s work may be difficult to quantify, but it can certainly be felt. To start to quantify this cognitive load, “Appointments Closed on Same Day” (%EncClosed) measures the percent of notes closed on the same day as the encounter. The burden experienced by clinicians from those notes closed the same day should be fleeting. However, while it shows a directionality with burnout, %EncClosed was not shown to carry the same significant association with burnout as CTCC in this study. The distinction between the two measures is in the cumulative cognitive load represented by CTCC. A clinician who completes the previous day’s notes the following morning might have a similar “%EncClosed” metric as someone who is chronically overwhelmed by documentation and requires a week to finish notes; the difference between the two physicians in their experience of burden, their CTCC metric, and likely their risk of burnout, is quite high, however. CTCC therefore intuitively measures the balance between job and personal demands and resources, serving as a warning sign when demands exceed resources or when these demands are unrealistic.

Notably, EHR-Time8 and Note-Time8 were not significantly associated with burnout in our study, suggesting that purely measuring time of interaction with the EHR is not sufficient to capture the dimensions of burnout. Although potentially useful in intervention design, activity-specific or schedule-specific time-based EHR use measures do not capture the heterogeneity of physicians’ responsibilities, work preferences, or total job burden, and may not be able to be used as a proxy for burnout. Minimizing CTCC may have benefits beyond burnout mitigation, reaching into clinical and financial domains. When a note remains incomplete or other aspects of care delivered are not documented in a timely fashion, the benefit of that information is not available for others who may need to care for the patient. From a revenue perspective, time-based billing practices for evaluation and management of ambulatory visits requires timely documentation for full insurance payment; time spent on patient care will only be reimbursed in full if it occurs on the day of service.^14^

As a harbinger and proxy for burnout, CTCC can be used to identify those at risk of burnout as well as to evaluate interventions designed to reduce burnout. High CTCC can both lead to burnout due to the metal load experienced by delaying chart completion, and be a proxy for burnout as a manifestation of the imbalance between resources and demands to complete one’s work and bear the weight of other life responsibilities. Health care systems can easily derive CTCC from widely available EHR-use timestamps to identify individuals or cohorts of providers who are burned out or at risk of burning out. Targeted interventions should be designed to better support these clinicians, as well as prevent others from reaching a tipping point. CTCC can then be followed over time to determine the impact of those interventions on workload burden and burnout.

Limitations of this study include a cross-sectional design which may not account for changes in either demands or resources over time. For this reason, CTCC is most useful when used to capture trends over time, whether for individual clinicians or clusters of clinicians within a healthcare system. Burnout scores themselves are subject to participant reactivity and participation bias. While trainees are especially vulnerable to burnout, they were not included in this analysis as closure of their documentation is dependent on attending preceptor sign-off. These results are derived from encounters that happened during the COVID-19 pandemic and reflect workflows that may be in flux. However, the point in time nature of measuring CTCC makes it an effective measure of current state as well as assessing impact of interventions over time. The rolling-window nature of CTCC allows for iteration in window length to define optimal duration over which to capture the measure. Future studies will explore the variation in time and predictors of CTCC.

Despite these limitations, this study includes data on EHR use patterns and burnout from a large, multispecialty academic medical center with results adjusted to account for potential confounders such as age, gender, and years of practice. Burnout was assessed administratively by the organization, not solely for the purpose of association with CTCC or EHR use measures, thus potentially lessening the influence of the purpose of the data collection on the results. The time spent on the EHR was directly derived from audit logs, eliminating the potential confounder of physician recall when estimating the time spent on specific EHR activities.

Critically, and distinct from other measures that account for EHR use alone, CTCC transcends individual practice patterns and accounts for the balance of demand and resource availability for the individual clinician. It is a measure that would be difficult to artificially skew or deflate in designing interventions due to the varied drivers of workload burden as measured by CTCC. As well, given the multifactorial nature of the drivers of burnout, process measures that account for EHR use alone may not sufficiently characterize burnout to serve as surrogates in evaluating interventions. More holistic metrics such as CTCC, which capture the interplay between the temporal dimensions of work and “life,” or non-work factors, are crucial as we seek to understand the causes of burnout. We posit this also allows CTCC to better serve as a predictor for burnout, as CTCC is measured over a period of time whereas any survey necessarily captures responses from a moment in time.

CTCC has the potential to identify clinicians at risk for burnout, while also serving as an evaluation metric of burnout interventions. CTCC is a measure of workload burden—when there is an imbalance between system and personal resources, and job and personal demands we would expect CTCC to increase; similarly, as CTCC increases, we have shown that it is more likely that someone will be burned out. CTCC is therefore both a driver of and proxy for burnout. CTCC can serve as an efficiently and rapidly measured outcome metric of wellness, not just a process metric on the road from intervention to vitality.

## Supplementary Material

ooae009_Supplementary_Data

## Data Availability

The data underlying this article cannot be shared publicly to maintain the privacy of clinicians in the study per institutional IRB requirement. Our dataset includes sensitive data such as individuals’ burnout survey responses.
