# Supplementary material for: Cumulated time to chart closure: a novel electronic health record-derived metric associated with clinician burnout
Source: JAMIA Open. 2024 Feb 8;7(1):ooae009. doi: 10.1093/jamiaopen/ooae009 (PMC10852987; doi:10.1093/jamiaopen/ooae009)
Supplement: ooae009_Supplementary_Data [file ooae009_supplementary_data.zip › Supp table 2_10.16.23.docx]

| **Supplementary Table 2**. Parameter estimates from final logistic regression model predicting burnout as outcome with Cumulated Time to Chart Closure and IB-Time_8_ | | | | |
| --- | --- | --- | --- | --- |
| **Independent Variable** | **Level**^†^ | **Reference**^†^ | **Odds Ratio (95% CI)** | ***P* value** |
| **Cumulated Time to Chart Closure** |  |  | 1.43 (1.02, 1.99) | 0.0363* |
| IB-Time_8_ |  |  | 2.10 (1.00, 4.41) | 0.0509 |
| **Age** |  |  | 1.02 (0.77, 1.34) | 0.9152 |
| **Race/Ethnicity** | Asian | White | 1.33 (0.70, 2.55) | 0.3865 |
|  | Under-Represented Minorities |  | 1.50 (0.68, 3.31) | 0.3209 |
|  | Unknown / Missing |  | 1.80 (0.60, 5.36) | 0.2945 |
| **Gender** | Male | Female | 1.15 (0.67, 1.99) | 0.6120 |
|  | Self-defined or missing |  | 0.72 (0.22, 2.38) | 0.5941 |
| **Academic Rank** | Associate Professor | Professor | 0.57 (0.19, 1.75) | 0.3285 |
|  | Assistant Professor |  | 1.04 (0.35, 3.11) | 0.9415 |
|  | Instructor |  | 1.05 (0.27, 4.06) | 0.9385 |
|  | Missing |  | 0.28 (0.06, 1.22) | 0.0893 |
| **Specialty** | Medical | Surgical | 1.32 (0.74, 2.37) | 0.3442 |
|  | Procedural |  | 1.89 (0.39, 9.25) | 0.4331 |

^†^For categorial variables

*Statistically significant
