# Supplementary material for: Cumulated time to chart closure: a novel electronic health record-derived metric associated with clinician burnout
Source: JAMIA Open. 2024 Feb 8;7(1):ooae009. doi: 10.1093/jamiaopen/ooae009 (PMC10852987; doi:10.1093/jamiaopen/ooae009)
Supplement: ooae009_Supplementary_Data [file ooae009_supplementary_data.zip › Supplementary Table 1_CTCC_10.16.23.docx]

| **Supplementary Table 1**. Parameter estimates from final logistic regression model predicting burnout as outcome with CTCC and WOW_8_ | | | | |
| --- | --- | --- | --- | --- |
| **Independent Variable** | **Level**^†^ | **Reference**^†^ | **Odds Ratio (95% CI)** | ***P* value** |
| **Cumulated Time to Chart Closure** |  |  | 1.38 (0.98, 1.94) | 0.0677 |
| **WOW_8_** |  |  | 1.76 (0.86, 3.56) | 0.1197 |
| **Age** |  |  | 0.99 (0.75, 1.30) | 0.9148 |
| **Race/Ethnicity** | Asian | White | 1.29 (0.67, 2.45) | 0.4452 |
|  | Under-Represented Minorities |  | 1.53 (0.69, 3.39) | 0.2973 |
|  | Unknown / Missing |  | 1.81 (0.62, 5.29) | 0.2808 |
| **Gender** | Male | Female | 1.15 (0.67, 1.99) | 0.6083 |
|  | Self-defined or missing |  | 0.71 (0.22, 2.28) | 0.5633 |
| **Academic Rank** | Associate Professor | Professor | 0.61 (0.20, 1.87) | 0.3873 |
|  | Assistant Professor |  | 1.09 (0.37, 3.26) | 0.8755 |
|  | Instructor |  | 1.15 (0.30, 4.43) | 0.8431 |
|  | Missing |  | 0.29 (0.07, 1.26) | 0.0995 |
| **Specialty** | Medical | Surgical | 1.38 (0.77, 2.46) | 0.2739 |
|  | Procedural |  | 1.94 (0.39, 9.67) | 0.4200 |

^†^For categorial variables

*Statistically significant
